# The impact of propagule pressure on whole community invasions in biomethane-producing communities

**DOI:** 10.1016/j.isci.2021.102659

**Published:** 2021-05-28

**Authors:** Pawel Sierocinski, Jesica Soria Pascual, Daniel Padfield, Mike Salter, Angus Buckling

**Affiliations:** 1 Environment and Sustainability Institute, Penryn Campus, University of Exeter, Penryn, UK; 2 AB Agri Ltd, Peterborough, UK

**Keywords:** Ecology, Microbiology, Applied microbiology

## Abstract

Microbes can invade as whole communities, but the ecology of whole community invasions is poorly understood. Here, we investigate how invader propagule pressure (the number of invading organisms) affects the composition and function of invaded laboratory methanogenic communities. An invading community was equally successful at establishing itself in a resident community regardless of propagule pressure, which varied between 0.01 and 10% of the size resident community. Invasion resulted in enhanced biogas production (to the level of the pure invading community) but only when propagule pressure was 1% or greater. This inconsistency between invasion success and changes in function can be explained by a lower richness of invading taxa at lower propagule pressures, and an important functional role of the taxa that were absent. Our results highlight that whole community invasion ecology cannot simply be extrapolated from our understanding of single species invasions. Moreover, we show that methane production can be enhanced by invading poorly performing reactors with a better performing community at levels that may be practical in industrial settings.

## Highlights

• Complex communities can successfully invade other communities
• Invasions change the community composition independently of propagule pressure
• Number of taxa invading increases with increased propagule pressure
• Larger propagule pressure impacts both the community structure and function

## Graphical abstract

**Figure:**
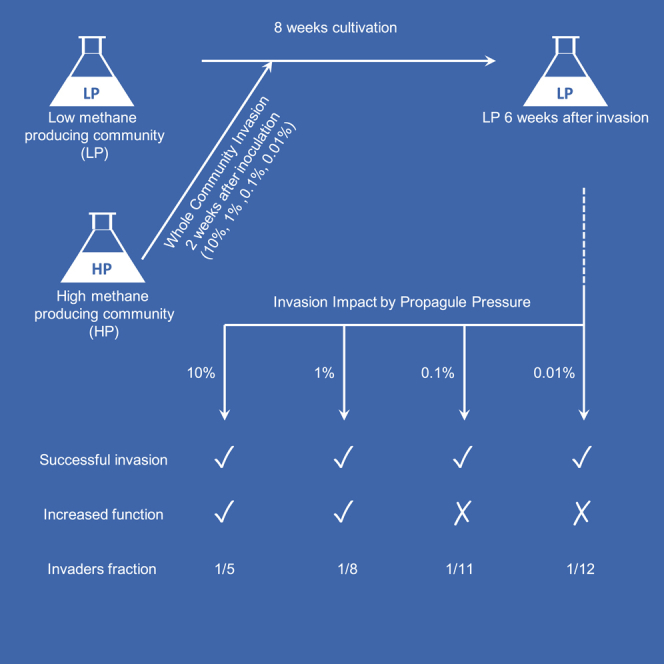

## Introduction

Plant and animal invaders can play a major role in the structure and function of natural ecosystems (Montoya et al., 2006; O'Dowd et al., 2003; Wardle et al., 2011). Microbial populations and communities, like those of plants and animals, are also geographically structured (DeLong et al., 2006; Martiny et al., 2006; Nemergut et al., 2013; Whitaker et al., 2003), suggesting an important role of microbial invasions in their formation. We know relatively little about the causes and consequences of microbial invasions (Mallon et al., 2015; Thakur et al., 2019), but there are clear parallels with the more extensive research on plant and animal invasion ecology (Eisenhauer et al., 2013; Hall et al., 2012; Lear et al., 2020; van Elsas et al., 2012). For example, resident diversity typically reduces invasion success (Hodgson et al., 2002; Levine and D'Antonio, 1999), while higher invader propagule pressure (the number of invading organism) (Acosta et al., 2015; Ketola et al., 2017; Lockwood et al., 2005) and disturbances (Lembrechts et al., 2016; Rivett et al., 2018) may promote invasion. However, there are also likely major differences between microbial and macrobial invader dynamics. Notably, microbes often invade as whole communities, rather than as single species (Bellucci et al., 2015; Castledine et al., 2020; Rillig et al., 2015). Leaves falling on the ground (Koide et al., 2005), the release of sewage into the rivers (Mansour et al., 2018) and fecal transplants (Gough et al., 2011) are examples of entire microbial assemblies arriving in a new ecosystem.

Here, we empirically address one of the simplest potential predictors of community invasion success: propagule pressure (Acosta et al., 2015; Ketola et al., 2017; Vila et al., 2019). Higher propagule pressure is predicted to increase invasion success by reducing stochastic loss of invaders. A larger community is also likely to be more diverse (Bell et al., 2005; Diamond and Mayr, 1976). High community diversity can promote whole community invasion (Rivett et al., 2018; Vila et al., 2019) because more diverse communities are both more likely to contain a particularly invasive species and show greater niche complementarity; the latter increasing positive co-selection between community members (Sierocinski et al., 2017; Tikhonov, 2016). Larger population sizes of invading species are also likely to result in invader populations adapting to new ecological conditions more rapidly (Lachapelle et al., 2015).

While higher propagule pressure is expected to increase invasion success of both single species and whole communities, the functional consequences of invasion may be less predictable for whole communities. The positive correlation between propagule pressure and species diversity means a successful invasion under low propagule pressure will result in a lower diversity of invaders than under high propagule pressure. The resultant absence of some rare invading taxa under low propagule pressure will likely have functional consequences: there is growing evidence that rare taxa play a crucial role in microbial community functions (Jousset et al., 2017; Rivett et al., 2018; Sierocinski et al., 2018). By contrast, a successful invasion by a single species might be expected to have similar consequences for community function regardless of propagule pressure.

Here, we use methanogenic communities to empirically investigate the relationships between whole community propagule pressure on invasion success and the resultant community structure and function. A key functional consequence of community invasions of methanogenic communities is likely to be increased methane production. This is because efficient resource use by communities—for which methane production is a proxy—is a predictor of successful community invasions (Sierocinski et al., 2017; Tikhonov, 2016). Given the importance of methane for both renewable energy and climate change (Monteux et al., 2018), understanding the invasion ecology of methanogenic communities is therefore of broad relevance. We attempted to invade a lower methane-producing community (LP) with a higher methane-producing community across 3 orders of magnitude of propagule pressure (~0.01%–10% of the resident community size), measuring invasion success, total community composition, and methane production after 6 weeks.

## Results

### Invasion success

**Figure 1:**
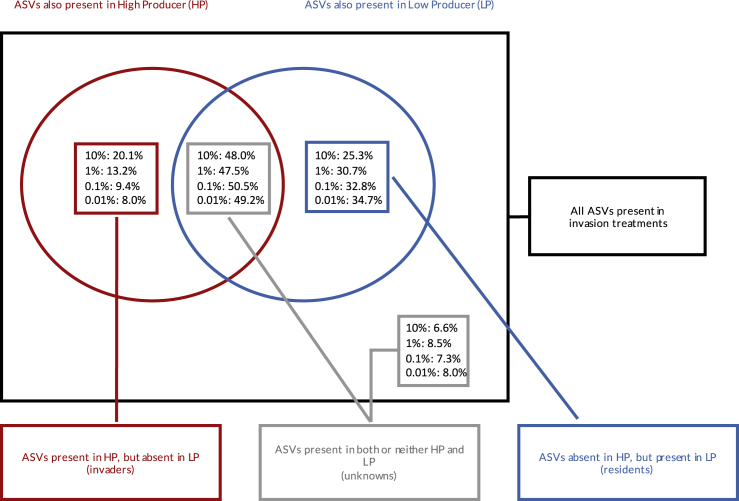
Schematic demonstrating how we attributed reads and ASVs to look at invader density and diversity We checked if the ASVs found in the invasion treatments were also present in the LP and HP pure communities. ASVs found in one of the pure communities were attributed as originating from that pure community. ASVs found in both or neither pure communities were not attributed.

**Figure 2:**
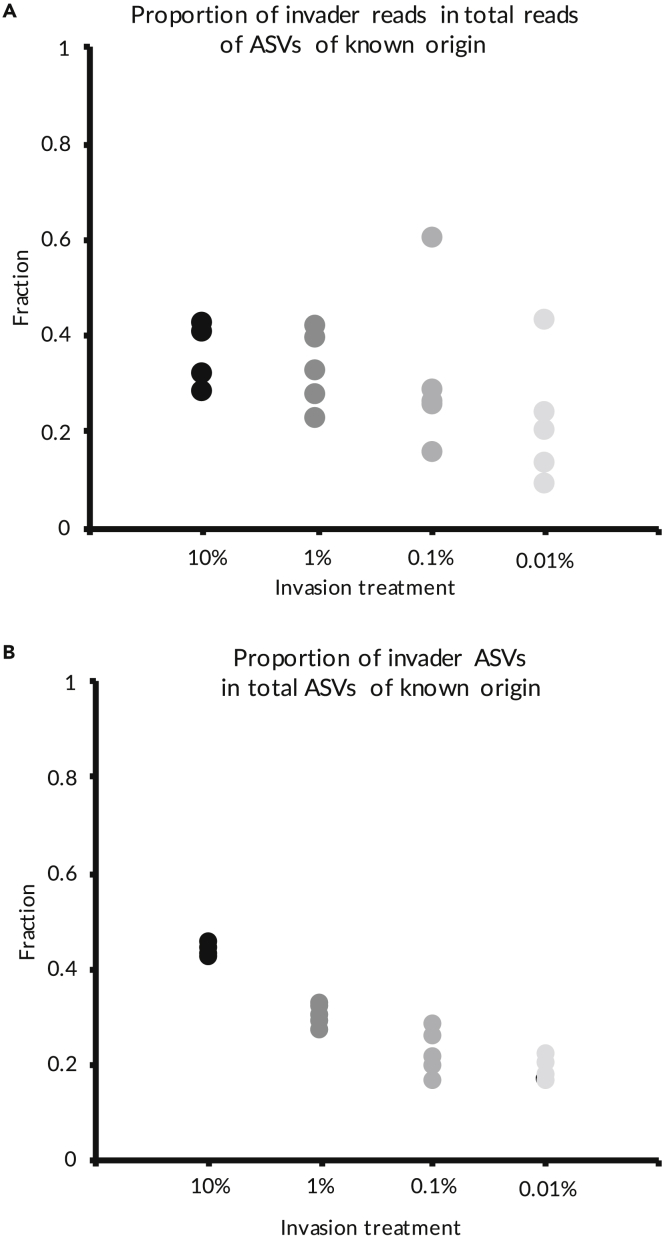
Invader abundance and diversity (A) Fraction of invader ASV reads in the total number of known origin ASV reads (invader abundance); (B) Fraction of invader ASVs in total known origin ASVs (invader diversity). Linked to Figure S1.

While only between 4 and 9% of all reads and between 40 and 47% of all ASVs in the invaded communities were attributable to only 1 community (Figure 1), the higher methane-producing (HP) community appeared to successfully invade the lower methane-producing (LP) community in all cases. The mean percentage of HP in total reads of attributable origin (Figure 2A) varied between 8.5% and 60.3%. The mean percentage of HP ASVs in total ASVs of attributable origin (Figure 2B) was between 16.3% and 46.6%. This means that even if all the non-attributed reads and ASVs originated from the LP community, a minimum of 0.7% and 7%, for total number of reads and numbers of ASVs, respectively, would still be attributable to the invading community (HP). These are much higher values than the lowest propagule pressure treatment (0.01%).

HP invasion success in terms of number of reads did not vary with propagule pressure (Figure 2B, F_1,18_ = 1.85, *P* = 0.19). However, the percentage of invading HP ASVs increased with propagule pressure (F_1,18_ = 88.2, *P* < 0.001). There was an average of 90 invader ASVs in the 10% propagule pressure treatment, 53 ASVs in 1% treatment, and 30 ASVs in 0.1% and 0.01% treatments. The invading ASVs were biased toward rarer taxa with respect to the composition of the entire invaded communities (Figure S1).

### Compositional changes following invasion

**Figure 3:**
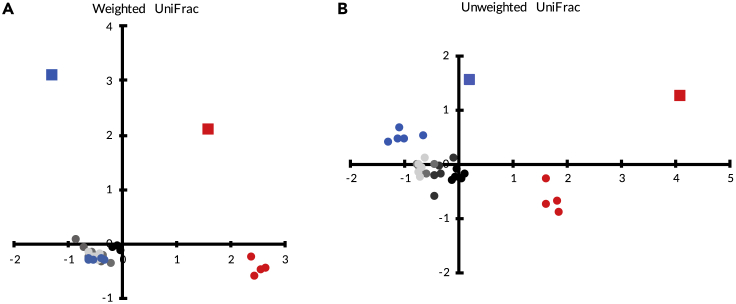
NMDS plots of beta diversity Weighted (A) and Unweighted (B) UniFrac of all the treatments. Red square, high producer ancestor; Blue square, low producer ancestor; Red circles, high producer; Blue circles, low producer; Black to light gray, invasion treatments; in order from darkest to lightest, 10%, 1%, 0.1%, 0.01%

The composition of most of the invaded communities resembled the uninvaded LP communities when considering the abundances of all ASVs found in the samples (Weighted UniFrac): only the 10% (and HP) treatments differed in composition from the LP community (Figure 3A, adonis2, F_5,23_ = 14.59 R^2^ = 0.76, *P =* 0.001, Pairwise adonis: *P* < 0.01). By contrast, all invaded communities were different from the LP community based on ASV presence and absence only (Unweighted UniFrac) (Figure 3B, adonis2, F_5,23_ = 7.76, R^2^ = 0.62, *P* < 0.001. Pairwise adonis: *P* = 0.002). Note that communities changed considerably from the ancestral LP and HP communities over the course of the experiment, largely because of ASV losses.

**Figure 4:**
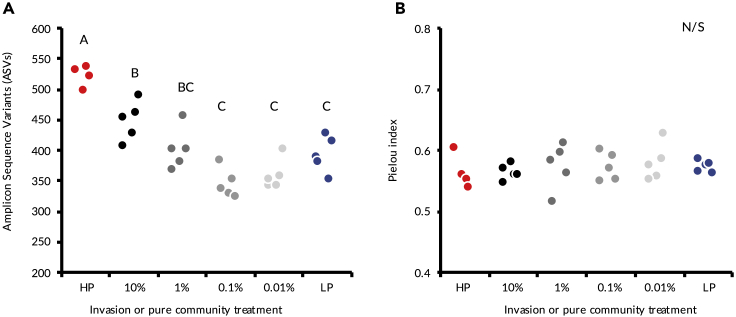
Richness and evenness of the treatments (A) Number of Amplicon Sequence Variants per sample (richness). (B) Pielou index (evenness).

We also determined how invasion affected total within-community diversity metrics. The HP community had significantly higher ASV richness than either the LP community or any of the invaded communities (Figure 4A ANOVA, F_5,23_ = 24.5, *P* < 0.001, Tukey-Kramer HSD: *P* < 0.01), and there was a positive relationship between the richness of the invaded LP communities and propagule pressure (F_1,27_ = 43.16, *P* < 0.001, R^2^ = 0.6). Species evenness (Pielou index) did not differ between the treatments (Figure 4B, ANOVA, F_5,23_ = 0.3, *P* = 0.91).

### Gas production

**Figure 5:**
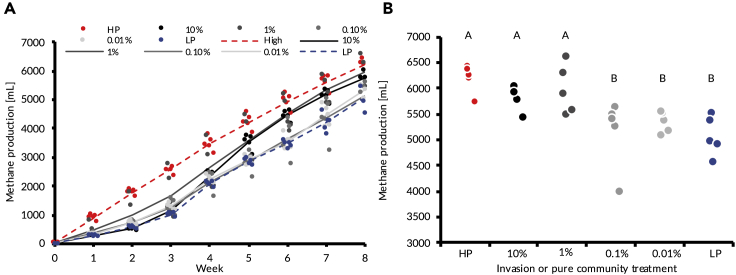
Methane gas production of the communities Mean gas production over time (A). Points are individual replicates, while lines show treatment means. Cumulative gas production at week 8 by treatment (B).

Total gas production of invaded communities increased with propagule pressure (Figure 5B, F_1,23_ = 10.23, *P* = 0.003 R^2^ = 0.28). This enhancement of gas production resulted in the 10% and 1% propagule pressure treatments resulting in gas production no different to the HP communities, while the 0.1% and 0.01% treatments produced significantly less gas than the HP communities (Figure 5A, Dunnett Test with HP as control, *P* < 0.05 for significant differences). Conversely, the 10% and 1% treatments produced more gas than the LP communities, while the 0.1% and 0.01% treatments and LP communities did not significantly differ in gas production (Figure 5A, Dunnett Test with LP as control, *P* < 0.05 for significant differences).

### Species analysis

The communities were composed mainly of *Firmicutes* and *Bacteroidetes* phyla, with methanogens dominated by acetoclasts (as opposed to hydrogenotrophs). Given the apparent “tipping point” in gas production between 0.1% and 1% propagule pressure communities, we determined if we could link this increase in function with particular taxa. We identified 44 ASVs (supplemental information) that were present in the 10% and 1% but not the 0.1% or 0.01% treatment, and further refined these to 13 ASVs that were detectable in >50% of the 10% and 1% replicates. These 13 ASVs were mainly in *Firmicutes*, with ASVs belonging to the *Ruminococcaceae* family being the most represented (4 ASVs). Aside from the Firmicutes, we found 2 abundant ASVs in the *Bacteroidetes* phylum from the *Porphyromonadaceae* family. One of those, a member of *Petrimonas* genus, was particularly numerous.

## Discussion

In contrast to our expectation, we found whole community invasion success of methanogenic communities to be independent of propagule pressure. Biogas production (a proxy for community productivity), was greater in the invaded communities (compared to the LP community), and increased with propagule pressure. The relationship was stepped: gas production of the resident community was not altered by 0.01% and 0.1% invasion but resembled that of the invading community with 1 and 10% invader doses.

The disparity between the different measures of invasion success (number of reads and number of taxa) is presumably because lower propagule pressure results in the loss of rare ASVs, while number of invader reads was mainly influenced by few high read ASVs. Either fewer invader ASVs were initially inoculated into the resident community, or the reduced population size of some ASVs resulted in stochastic loss during invasion. In contrast to recent work using synthetic communities (Goldford et al., 2018), these results suggest that rare taxa did not significantly contribute to the success of the invading community as a whole as a consequence of co-selection between community members.

The loss of rare taxa with lower initial invader dose can also explain patterns of gas production. Increasing propagule pressure from 0.1 to 1% led to approximately 40 more ASVs successfully invading and a marked increase in gas production. This is consistent with previous findings that reducing number of rare taxa through culture dilution reduces biogas production (Sierocinski et al., 2018). Note that the increase in gas production with invader dose can't be explained by key resources carried over from the media of the invading community, because there was a lag of approximately 4 weeks before gas production by the invaded communities started to increase.

While this work demonstrates that communities are capable of invading other communities from very rare, invading communities did not dominate the final community structure. Both theory (Tikhonov, 2016) and our previous experiments (Sierocinski et al, 2017, 2018) using methanogenic communities suggest that the most productive communities will dominate within a mixture of communities. However, in our previous study, unlike here, the communities were mixed at one to one ratio. The lack of domination of the invading communities in this study cannot be explained by invaders simply not having reached equilibrium conditions. The proportion of invader reads was the same regardless of invasion treatment, meaning that communities likely stabilized before the end point of the experiment. This suggests instead that there may have been a resident advantage through positive frequency or density dependence (a priority effect; e.g. see (Connell and Slatyer, 1977; Fukami, 2015)), as might arise through coevolved mutualistic interactions present in methanogenic communities (Hillesland and Stahl, 2010).

Given the stepped increase in gas production with invader dose increase, the taxa responsible for the increased gas production were presumably those present in the 10% and 1%, but not the 0.1% and 0.01%, invasion treatments. Thirteen ASVs fitted that criterion and were present in more than half of the 10% and 1% invasion replicates. Only seven of them could be identified to the family level. Four of the taxa present in the majority of the treatments belonged to *Ruminococcaceae,* two to *Porphyromonadaceae* and one to *Syntrophomonadaceae* families, and are most likely involved in hydrolysis, protein metabolism and direct interspecies electron transfer (Grabowski et al., 2005; Vartoukian et al., 2007; Wang et al., 2020; Zhang et al., 2017). While the first is not surprising, as the medium used in experiments is predominantly made from complex sugars, the other two point to interesting possibilities. Selection for organisms efficient at protein metabolism functions suggests a link between community function and nutrient scavenging. The abundance of *Petrimonas* ASV from *Porphyromonadaceae* family could be linked to facilitating electron transfer between species and use of acetate in biogas production (Wang et al., 2020). Given the dominance of acetoclastic methanogens in our bioreactors, this particular ASV is the most likely driver of increased biogas yield in the invaded communities.

Our results are most relevant to communities experiencing environmental change, where an invading community is potentially better adapted to at least some local conditions than are the residents. While invasion in this context may rapidly lead to restoration of a locally adapted community, our results suggest there may not be a concomitant restoration of function. Our results also suggest the potential for a novel approach of enhancing methane production in industrial settings. We have previously established that seeding an Anaerobic Digester from a mixture of starting communities will, on an average, result in improved gas production because the most productive communities tend to dominate (Sierocinski et al., 2017). However, improving performance of existing reactors inevitably requires successful invasion by a smaller community than the resident. We show that invader dose as low as 0.01% can successfully invade. While the lower initial invader dose did not result in increased gas production, increasing absolute size of invader communities could overcome the problem associated with loss of rare taxa.

### Limitations of the study

Community invasions may be less likely where resident communities are more locally adapted in which case residents will likely have an advantage (Fukami, 2015). Our manipulation of propagule pressure necessarily altered diversity of invaders, as would be the case in natural invasions. This could have made it difficult distinguishing the effect of number and diversity invaders. While our sequencing depth was relatively large, we work on non-defined communities. This means not all of the diversity within the communities might have been captured. Further work is also needed to determine if other types of communities, such as soil (Calderón et al., 2017; Gravuer and Scow, 2021), would show similar patterns. Finally, it is unclear if our results would scale up to an industrial setting.

## STAR★Methods

### Key resources table

**Table:** 

REAGENT or RESOURCE	SOURCE	IDENTIFIER
**Chemicals, peptides, and recombinant proteins**		

Bacto™ Protease Peptone no. 3	Thermo Scientific	211693

**Critical commercial assays**		

MP Biomedicals™ FastDNA™ SPIN Kit for Soil	Fisher Scientific	11492400
Agencourt AMPure XP PCR	Beckman Coulter	A63881

**Deposited data**		

Sequencing raw data from this experiment	ENA	PRJEB43371

**Experimental models: Organisms/strains**		

High methane-producing microbial community	Agricultural AD plant, Cornwall, this study	N/A
Low methane-producing microbial community	Waste Water AD plant, Cornwall, this study	N/A

**Software and algorithms**		

MiSeq Control Software 2.2.0	Illumina	
RTA 1.17.28	Illumina	

**Other**		

Automated Methane Potential Test System	Bioprocess Control Sweden AB	01-0000-02

### Resource availability

#### Lead contact

Any further requests relating to the article, including the requests for reagents or resources should be addressed to the lead contact, Pawel Sierocinski (p.sierocinski@exeter.ac.uk)

#### Materials availability

This study did not generate any new materials

#### Data and code availability

Sequencing data generated during the course of the experiments described in this article were deposited in ENA (accession no. PRJEB43371).

#### Experimental model and subject details

Methane-producing communities used in this study were collected at two Anaerobic Digestion plants in Cornwall, United Kingdom. The communities were grown in the lab using the Automated Methane Potential Test System (AMPTS, Bioprocess Control Sweden AB). The microbial communities were stored at 4°C prior to use, and then grown at 37°C in 500 ml bottle-fermenters (Duran) in the AMPTS.

### Method details

#### Experimental design

We used communities isolated from two anaerobic digester (AD) plants from the UK South West region. Before the experiment, we ran a pilot experiment for 4 weeks to measure methane production of each community. The stock communities were stored at 4°C for the duration of the experiment. We selected the community that produced the least methane of the two (Low Producer, LP) and cultivated 25 LP replicate fermenters using this community and 5 replicate fermenters of the community that produced more methane (High Producer; HP) in fermenters for two weeks to allow them to pre-adapt to experimental conditions. After two weeks, we invaded 20 of the LP replicates with either 10%, 1%, 0.1%, and 0.01% (by volume) of one of the HP replicates (5 replicates for each level of propagule pressure), while 5 LP replicates and 5 HP replicates were left undisturbed. The invaded LP communities and pure LP and HP communities were cultivated for an additional 6 weeks post-invasion, with gas production measured throughout and community composition determined at the beginning and end of the experiment.

#### Cultivation and gas measurement

All replicates were grown in 500 mL bottles (Duran, 600mL total volume with headspace) using Automated Methane Potential Test System (AMPTS, Bioprocess Control Sweden AB) (Sierocinski et al., 2017) to measure CO_2_-stripped biogas (Biogas) production. The pilot experiment and the pure LP and HP communities were started with 200 mL of the community. The 10% Invasion replicates were initiated with 180 mL of LP community and invaded with 20 mL of HP community after 2 weeks, while 1%, 0.1%, and 0.01% treatments were initiated with 198 mL of LP and invaded with 2 mL of either pure or HP, or HP diluted 10 or 100-fold with ddH_2_O, as appropriate. Each community was supplemented with 0.2 mL 1000 x concentrated trace metal solution (1 g L^-1^ FeCl_2_ 4H_2_O, 0.5 g L^-1^ MnCl_2_ 4H_2_O, 0.3 g L^-1^ CoCl_2_ 4H_2_O, 0.2 g L^-1^ ZnCl_2_, 0.1 g L^-1^ NiSO_4_ 6H_2_O, 0.05 g L^-1^ Na_2_MoO_4_ 4H_2_O, 0.02 g L^-1^ H_3_BO_3_, 0.008 g L^-1^ Na_2_WO_4_ 2H_2_O, 0.006 g L^-1^ Na_2_SeO_3_ 5H_2_O, 0.002 g L^-1^ CuCl_2_ 2H_2_O) at the start of the experiment. In both the pilot and main experiments, all communities were fed weekly, starting on t_0_ with 2g of feed composed of 3.53% casein, 1.17% peptone, 1.17% albumen, 47.07% dextrin, and 47.07% sucrose (all compounds Sigma, except for Bacto™ protease peptone no. 3 , Thermo Fisher). The feed was suspended in 18 mL sterile water.

#### DNA extractions and sequencing

DNA was extracted and sequenced as described previously (Sierocinski et al., 2018). We extracted DNA using FastDNA™ SPIN Kit for Soil (MP). We confirmed the quality of extractions by electrophoresis on a 1% agarose gel, and we quantified it using dsDNA BR (Qubit). We constructed the 16S rRNA gene libraries using primers designed to amplify the V4 region (Kozich et al., 2013) and multiplexed. We generated amplicons using a high-fidelity polymerase (Kapa 2G Robust) and purified them using the Agencourt AMPure XP PCR purification system. They were quantified fluorometrically (Qubit, Life Technologies). We pooled the amplicons in equimolar concentrations based on Qubit results. We diluted the amplicon library pool to 2 nM in sodium hydroxide and transferred 5 μL into 995 μL HT1 (Illumina) to give a final concentration of 10 pM. We spiked 600 μL of the diluted library pool with 10% PhiX Control v3 on ice before loading into the Illumina MiSeq cartridge following the manufacturer’s instructions. The sequencing chemistry utilized was MiSeq Reagent Kit v2 (500 cycles) with run metrics of 250 cycles for each paired-end read using MiSeq Control Software 2.2.0 and RTA 1.17.28. One of the samples from HP treatment failed to sequence and is not included in the analysis. The number of reads per successful sample ranged from 350 000 to 500 000. Sequence data is available on the European Nucleotide Archive under the accession number PRJEB43371.

### Quantification and statistical analysis

Sequencing data were analyzed in R (v 3.3.2) using the packages *‘dada2’* (Callahan et al., 2016), ‘*phyloseq’* (McMurdie and Holmes, 2013), ‘*vegan*’ (Oksanen et al., 2007) and ‘*pairwiseAdonis*’ (Martinez Arbizu, 2020). We used a full-stack workflow to estimate error rates, inferred and merged sequences, constructed a sequence table, removed chimeric sequences, and assigned taxonomy to each amplicon sequence variant (ASV) using the Greengenes database (DeSantis et al., 2006) following the published pipeline (Callahan et al., 2016). The data was not rarefied as the number of reads obtained from each sample did not vary by much and was large in all cases while rarefaction could result in information loss (McMurdie and Holmes, 2014). We estimated the phylogenetic tree using the R package ‘*phangorn*’, (Schliep, 2011) where we constructed a neighbor-joining tree, and then fit a Generalized time-reversible with Gamma rate variation maximum likelihood tree using the neighbor-joining tree as a starting point. We used UniFrac distance (weighted and unweighted) as our measure of compositional dissimilarity between the communities (Lozupone and Knight 2005). In case of alpha diversity, we calculated species richness and evenness (Pielou, 1966).

To establish if the invasions were successful, we needed to determine if organisms from the HP treatment managed to establish themselves in the invasion treatment. The majority of ASVs were shared between HP and LP samples (Figure 1) making it difficult to discriminate relative community contributions using previous statistical methods (Sierocinski et al., 2017). Instead we subset the 16S sequencing data. For each invaded community, we determined the ASVs of attributable origin: those that were present in the endpoint of only one of the pure community treatments. ASVs found in LP community, but not in HP were attributed to LP and vice versa. ASVs present in both or neither pure community treatments were excluded. Invasion success was then estimated in two ways: as the proportion of ASV reads of HP origin in total reads (proxy for the fraction of successfully invaded individuals) or as a proportion of ASVs of HP origin in all ASVs (proxy for fraction of species that successfully invaded the community). Separate linear models were used to determine whether final invader number or invader diversity was altered by initial invader abundance, where invader abundance was treated as a categorical predictor. Model selection was done using likelihood ratio tests and post-hoc pairwise comparisons were between individual treatments.

When looking at different community composition between treatments and pure LP and HP communities, we used all of the 16S sequencing data including all ASVs present in single treatments and pure LP and HP communities. We used a single permutational ANOVA to look at an overall difference in community composition between treatments, and used pairwise permutational ANOVA to see which treatments were driving any observed pattern. Differences in alpha diversity and evenness were tested using linear models, with number of ASVs (richness) or Pielou’s evenness as the response variable and treatment (invasion density or pure LP/HP community) as the predictor variable. Model selection and post-hoc comparisons were done as above. Similarly, gas production was compared using linear models, with gas production as a response variable and treatment (invasion density or pure LP/HP community) as the predictor variable. We used Dunnett’s Test to compare the invasion treatments to pure LP or pure HP gas production only, thus avoiding multiple comparisons between the invasion treatments. Model selection and post-hoc comparisons were done as above.

## Footnotes

Supplemental information can be found online at https://doi.org/10.1016/j.isci.2021.102659.
