# Supplementary material for: The impact of propagule pressure on whole community invasions in biomethane-producing communities
Source: iScience. 2021 May 28;24(6):102659. doi: 10.1016/j.isci.2021.102659 (PMC8192723; doi:10.1016/j.isci.2021.102659)
Supplement: Document S1. Figure S1 [file mmc1.pdf]

iScience, Volume *24*

Supplemental information

The impact of propagule pressure on whole community invasions in biomethane-producing communities

Pawel Sierocinski, Jesica Soria Pascual, Daniel Padfield, Mike Salter, and Angus Buckling

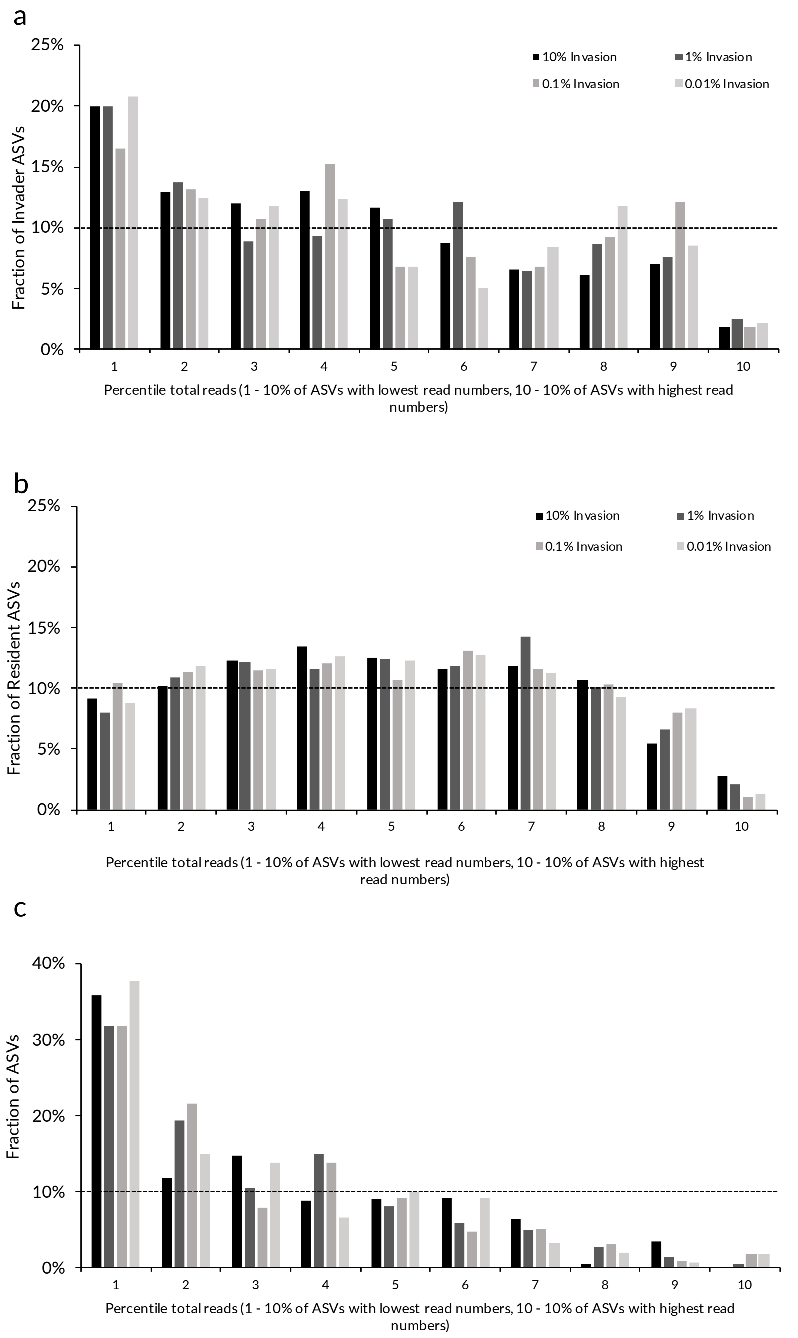

Supplemental Figure S1: **Abundance of invader and resident ASVs, related to Figure 2.** Fraction of invader ASVs (a), resident ASVs (b) and ASVs present in neither HP or LP samples (c) in each decile of total ASV by read number for each treatment level.
